# Reduction in psychotic symptoms as a predictor of patient satisfaction with antipsychotic medication in schizophrenia: Data from a randomized double-blind trial

**DOI:** 10.1186/1471-244X-6-45

**Published:** 2006-10-20

**Authors:** Georges M Gharabawi, Andrew Greenspan, Marcia FT Rupnow, Colette Kosik-Gonzalez, Cynthia A Bossie, Young Zhu, Amir H Kalali, A George Awad

**Affiliations:** 1 Medical Affairs, Janssen Pharmaceutica Inc., Titusville, NJ, USA; 2 Johnson & Johnson Pharmaceutical Research and Development, Titusville, NJ, USA; 3 Outcomes Research, Ortho-McNeil Janssen Scientific Services, L.L.C., Titusville, NJ, USA; 4 Quantitative Methodology, Ortho-McNeil Janssen Scientific Services, L.L.C., Titusville, NJ, USA; 5 Quintiles CNS Therapeutics, San Diego, California, USA; 6 University of California, San Diego, California, USA; 7 University of Toronto, Toronto, Ontario, Canada

## Abstract

**Background:**

Patient satisfaction with antipsychotic treatment is important. Limited evidence suggests that satisfaction is associated with symptom improvement and compliance. Predictors of patient satisfaction with antipsychotic medication were examined in a study of patients with a recent exacerbation of schizophrenia.

**Methods:**

Data are from a randomized, double-blind trial comparing risperidone (n = 152), quetiapine (n = 156), and placebo (n = 73). Medication Satisfaction Questionnaire (MSQ) was completed after 14 days of treatment and after 6 weeks at last study visit.

**Results:**

Medication satisfaction at both time points was significantly associated in multiple regression analysis with improvement on 3 Positive and Negative Syndrome Scale (PANSS) factor scores (positive symptoms p < .01; uncontrolled hostility/excitement, p < .0005; anxiety/depression, p < .04) and treatment with risperidone (p < .03); at day 14, significant association was also found with older age (p = .01). At both time points, predictor variables explained over 30% of the variance in medication satisfaction. Change in Hamilton Depression Scale, prolactin levels, sex, and reported adverse events of extrapyramidal symptoms, sedation, and movement disorders were not significant predictors of satisfaction. Lower level of medication satisfaction at day 14 was associated with earlier discontinuation in the trial at week 6 end point. A focused principal components analysis of PANSS factors and MSQ suggested that medication satisfaction relates to 3 groups of factors in descending order of magnitude: lower levels of (a) uncontrolled hostility/excitement, (b) positive symptoms, and (c) negative symptoms, disorganized thoughts, and anxiety/depression.

**Conclusion:**

Results give further support that treatment satisfaction is positively associated with symptom improvement, particularly psychotic symptoms, and suggest that satisfaction may also be related to compliance, as those who were more satisfied remained in the trial for a longer period of time.

**Trial registration number:**

Trial registration number NCT00061802

## Background

Patient satisfaction with antipsychotic treatment is an important outcome. There is limited evidence suggesting that it is positively associated with compliance [1], improved clinical outcomes [2-6], and quality of life [7]. Yet there is a lack of prospective studies examining the association of patient satisfaction, medication compliance, and treatment outcomes [8].

While considerable attention has been given to the efficacy and safety of second-generation antipsychotics, little attention in clinical trials has been given to medication compliance, subjective tolerability, and satisfaction with treatment [9,10]. There are some data from naturalistic studies suggesting greater satisfaction among patients treated with second-generation than first-generation antipsychotic medications [11,12]. It is surprising that patient satisfaction, which may be a key advantage of second-generation antipsychotics, has not received adequate research attention. This has led to the recognition that there is a need for well-designed studies of treatment satisfaction of second-generation antipsychotic medications before firm conclusions can be reached [13]. In the current study, we examined predictors and consequences of patient satisfaction with atypical antipsychotic medication in a study of patients with a recent exacerbation of schizophrenia treated with risperidone, quetiapine, or placebo.

## Methods

### Study design

Data are from a 2-phase, double-blind, international, 6-week study conducted at 30 sites. The safety and efficacy results and methodology are reported elsewhere [14]. Inpatients with schizophrenia or schizoaffective disorder with a recent exacerbation of psychotic symptoms were randomly assigned to receive risperidone, quetiapine, or placebo in a 2:2:1 ratio. Patients were treated with risperidone, quetiapine, or placebo monotherapy for the first 2 weeks; during the subsequent 4 weeks, investigators were permitted to prescribe additional psychiatric medications as necessary. Study medications were increased from days 1 to 5 according to a fixed schedule. Target doses at day 5 were 4 or 6 mg/day of risperidone and 400 or 600 mg/day of quetiapine. On day 8, the dose of quetiapine could be increased, in a blinded fashion, to 600 or 800 mg/day. Patients were maintained on their day-8 dose for the remainder of the study. Mean doses at day 14 were 4.7 ± 0.9 mg/day of risperidone and 579.5 ± 128.9 mg/day of quetiapine. Dosing regimens for risperidone and quetiapine were in accordance with the prescribing information for each drug and also reflected clinical and research practices for treating patients with acute exacerbations of schizophrenia [15,16].

The trial was conducted in accordance with current International Conference on Harmonization-Good Clinical Practice guidelines and the Declaration of Helsinki and its subsequent revisions. All patients deemed competent by the investigator provided written informed consent prior to study participation. If a patient was deemed not legally competent, then consent was obtained from the patient and an authorized representative. Ethical approval was obtained by Institutional Review Boards at each investigators site.

Exclusion criteria included a co-morbid Axis I diagnosis (with the exception of substance abuse/dependence), borderline personality disorder, mental retardation, or a clinically significant medical illness. Also excluded were patients who had received risperidone or quetiapine within 7 days of baseline, clozapine within 60 days, or depot antipsychotics or electroconvulsive therapy within defined time periods.

Baseline characteristics were similar in the 3 treatment arms [14]. The mean (± SD) age of patients was 34.8 ± 9.7 (median 35; range 18–63) years, and 60% were male. The mean (± SD) baseline Positive and Negative Syndrome Scale (PANSS) was 95.8 ± 18.5 and Clinical Global Impressions (CGI)-Severity was 5.4 ± 0.5. Eighty-seven percent of the patients completed the day 14 visit and 75% completed day 42.

### Efficacy and safety assessment

Assessments were conducted on days 1, 3, 5, 7, 9, 14, 21, 28, and 42. Efficacy measures included the PANSS [17]; the 17-item Hamilton-Depression Scale (HAM-D) [18]; and the CGI-Severity and CGI-Change scales [19]. Safety measures included the Simpson Angus Scale (SAS) [20] and the Barnes Akathisia Scale (BAS) [21], which were administered at baseline, day 14, 28, and 42. Reports of adverse events were collected at all visits, and laboratory assessments (including prolactin) were performed at baseline and days 14 and 42.

### Medication satisfaction

Patient satisfaction with the study medication was assessed using the Medication Satisfaction Questionnaire (MSQ) a 1-item global patient-rated scale. Specifically, patients were asked to respond on a 7-point scale, ranging from extremely dissatisfied (1) to extremely satisfied (7), to the following: "The way you feel about taking your study medication is". The MSQ was derived from work using a much longer and more detailed published scale [22] and is similar to items included in other scales [3]. There has been much debate in the field regarding the correct methodological approach for measuring patient satisfaction and no consensus has yet been reached. It has been argued that lengthier scales typically contain a large number of items that are irrelevant and thus are more susceptible to providing inaccurate results. Longer instruments of patient satisfaction have generally not demonstrated better validity than 1-item global ratings [23]. This is of particular relevance for acutely severely ill psychotic patients who could not reasonably be expected to complete a lengthy patient-rated instrument.

### Data analysis

We attempted to predict medication satisfaction at days 14 and 42 using univariate linear regression followed by step-wise multiple linear regression. The predictor variables chosen were change from baseline in symptoms (5 PANSS factor scores [24] and HAM-D scale), treatment regimen (risperidone, quetiapine, or placebo), reported adverse events of extrapyramidal symptoms (EPS) or sedation, movement disorders (BAS, SAS), prolactin levels, age, and sex. We also examined whether greater initial treatment satisfaction as measured at day 14 was associated with time in trial to end point. This was done by computing the mean time (in days) in trial for each response category on the MSQ.

Finally, to understand the relationship between psychotic symptomatology and medication satisfaction we examined the association of the MSQ and the 5 PANSS factors [24]. This was done first by examining the correlations of the MSQ and PANSS factors. To graphically represent and examine the relationship between Medication Satisfaction and the five PANSS factors we used focused principal components analysis (PCA) [25]. Focused PCA is a special type of PCA designed to describe and understand relationships between a set of quantitative variables, with a particular interest in the dependencies of one variable, in this case MSQ, with the others, in this case the PANSS factors. The relationships between nondependent variables are interpreted as in a PCA: correlated variables are close or diametrically opposite (for negative correlations); independent variables make a right angle with the origin. Focused PCA was conducted using R (version 2.0.1) software, module PSY (version 0.6).

## Results and Discussion

As shown in Table 1, medication satisfaction at both time points was significantly associated with improvement (change from baseline to each time point) on 3 PANSS factor scores (positive symptoms, uncontrolled hostility/excitement, anxiety/depression), treatment with risperidone, and at day 14 also higher age. At both time points, these predictor variables explained over 30% of the variance in medication satisfaction in a multiple regression analysis. Change in HAM-D scale, prolactin levels, sex, and reported adverse events of EPS, sedation, and movement disorders (BAS, SAS) were not significant predictors of satisfaction.

**Table 1:** Predictors of patient satisfaction with antipsychotic medication

	Day 14					Day 42 (end point)				
	Univariate regression			Step-wise multiple regression R^2 ^= .31		Univariate regression			Step-wise multiple regression R^2 ^= .33	

	T =	p =	R^2^	F =	p =	T =	p =	R^2^	F =	p =

Symptom change PANSS factors Positive	-10.11	<.0001	.26	21.98	<.0001	-9.40	<.0001	.23	6.39	.01
Negative	-5.22	<.0001	.11			-5.69	<.0001	.12	3.63	.06
Disorganized thoughts	-5.89	<.0001	.13			-5.64	<.0001	.12		
Hostility/excitement	-9.25	<.0001	.23	14.11	.0002	-8.69	<.0001	.21	12.46	.0005
Anxiety/depression	-5.80	<.0001	.13	4.03	.04	-7.43	<.0001	.17	16.85	<.0001
HAM-D	-5.33	<.0001	.11		NS	-5.06	<.0001	.11		NS
Adverse events Prolactin level	.439	.66	.04			-.666	.50	.04		NS
EPS reported	-.95	.34	.04	2.89	.09	.60	.55	.04		NS
Sedation reported	-1.21	.23	.05			1.91	.06	.05		NS
BAS global (present)	-1.42	.16	.05			-.88	.38	.04		
SAS total (present)	-.50	.62	.04			-.25	.80	.04		
Demographics Age	1.84	.07	.05	6.17	.01	.58	.56	.04		
Gender (male)	.46	.64	.04			.37	.71	.04		
Antipsychotic use		NA*				-2.69	.007	.06	8.03	.004
Risperidone	3.48	.0006	.04	4.80	.03	3.06	.002		4.94	.027
Quetiapine	1.15	.25	.04	.60	.44	.83	.41		.00	.98

* Antipsychotic use appeals after 14 days.

Abbreviations: PANSS, Positive and Negative Syndrome Scale; HAM-D, Hamilton Depression Scale; NS, not significant; EPS, extrapyramidal symptoms; BAS, Barnes Akathisia Scale; SAS, Simpson Angus Scale.

**Figure 1:**
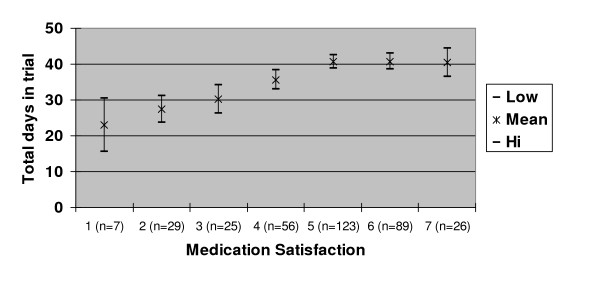
Mean (95% confidence interval) time (days) in trial by Medication Satisfaction after 14 days of treatment (1 very dissatisfied; 7 very satisfied).

Figure 1 shows the mean time (in days) in trial until week 6 end point by medication satisfaction after 14 days of treatment. Patients who were satisfied clearly remained in the study significantly longer than those who were not satisfied.

**Figure 2:**
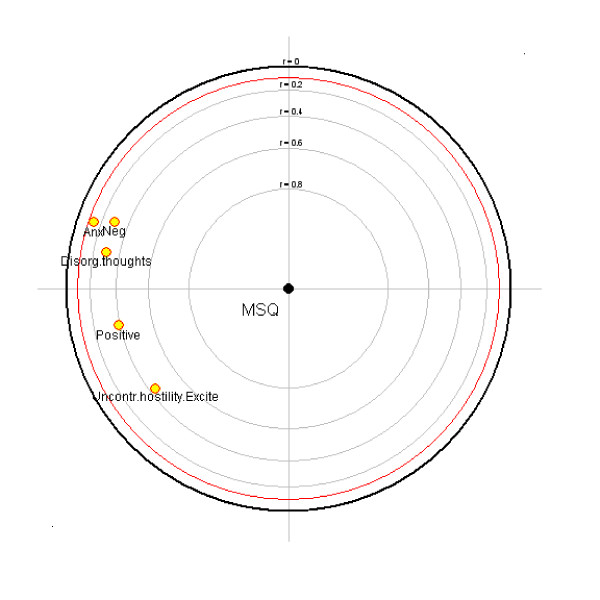
Focused principal components analysis of MSQ and PANSS factors. Abbreviations: MSQ, Medication Satisfaction Questionnaire; PANSS, Positive and Negative Syndrome Scale. As the rings get closer to the center they reflect a higher correlation with MSQ.

The correlation of the PANSS factors and MSQ (Table 2) and the focused PCA (Figure 2) suggest that medication satisfaction relates to 3 groups of factors in descending order of magnitude: lower levels of (a) uncontrolled hostility/excitement, (b) positive symptoms, and (c) negative symptoms, disorganized thoughts, and anxiety/depression. In the focused PCA (Figure 2) as the rings get closer to the center they reflect a higher correlation with the MSQ. The positive symptoms factor and uncontrolled hostility/excitement were the most closely correlated with the MSQ, followed by negative symptoms and disorganized thoughts, with little association with anxiety/depression.

## Conclusion

The results give further support that treatment satisfaction is positively associated with symptom improvement, particularly psychotic symptoms. The results also suggest that treatment satisfaction may also be related to compliance, since patients who were more satisfied remained in the trial for a longer time. A single-item measure of patient satisfaction was sensitive to clinical improvement and adherence. Patient-reported outcomes, such as satisfaction with medications, offer a way of measuring both efficacy and tolerability features of treatments from a patient perspective. As rated by the MSQ, data suggest that the significantly greater patient satisfaction with risperidone than quetiapine or placebo may be related to the greater symptom reduction associated with risperidone. This is suggested by both regression models in which a reduction in psychotic symptoms consistently emerged as a significant predictor of medication satisfaction. Further, in the multiple regression model, treatment with risperidone, but not quetiapine, and increasing age were also predictive of medication satisfaction. Our finding that patient satisfaction is related to improvement of positive symptoms and not negative symptoms might be related to the duration of the trial. As was the case in this trial, within 6 weeks, positive symptoms usually decline much more than negative or other symptoms. While certain safety issues are often cited as limiting factors for patient acceptability, neither movement disorders measures nor prolactin elevation was predictive of medication satisfaction.

Future studies of second-generation antipsychotic medications should routinely include measures of medication satisfaction. Unlike the current study, in which the measure was only administered at 2 time points, consideration should be given to measuring satisfaction each time efficacy is measured. With the advent of yet improved formulations and long-acting medications designed to improve compliance, medication satisfaction will be an outcome of major importance.

## Competing interests

GG declares he is an employee of Janssen, the company which funded the research, manuscript development, and the journal's article processing charge. GG is a J&J stockholder.

AG declares he is an employee of Johnson & Johnson Pharmaceutical Research and Development. AG is a J&J stockholder.

MR declares she is an employee of Janssen, the company which funded the research, manuscript development, and the journal's article processing charge. MR is a J&J stockholder.

CKG declares she is an employee of Janssen, the company which funded the research, manuscript development, and the journal's article processing charge. CKG is a J&J stockholder.

CB declares she is an employee of Janssen, the company which funded the research, manuscript development, and the journal's article processing charge. CB is a J&J stockholder.

YZ declares he is an employee of Janssen, the company which funded the research, manuscript development, and the journal's article processing charge. YZ is a J&J stockholder.

AK declares he is on the Janssen Speakers Bureau

AGA declares that he has no competing interests

## Authors' contributions

GG: Study conception and design, analysis plan, interpretation of data, input for manuscript revision.

AG: Acquisition of the data, analysis plan, interpretation of data, drafting and revising the manuscript.

MFTR: Study design, interpretation of data, input for manuscript revision.

CKG: Study design, acquisition of the data, interpretation of data, input for manuscript revision.

CB: Analysis plan, interpretation of data, drafting and revising the manuscript.

YZ: Analysis, input for manuscript revision.

AHK: Interpretation of data, input for manuscript revision.

AGW: Interpretation of data, input for manuscript revision.

All authors read and approved the final manuscript.

**Table 2:** Correlation of PANSS factors (change from baseline score) and medication satisfaction at day 14 (n = 381)

	r =	p <
Uncontrolled hostility/excitement	-.44	.00001
Positive symptoms	-.39	.00001
Negative symptoms	-.30	.00001
Disorganized thoughts	-.30	.00001
Anxiety/depression	-.14	.015

Abbreviations: PANSS, Positive and Negative Syndrome Scale.

## Pre-publication history

The pre-publication history for this paper can be accessed here:


